# Structure and function of bacteriophage CBA120 ORF211 (TSP2), the determinant of phage specificity towards *E. coli* O157:H7

**DOI:** 10.1038/s41598-020-72373-0

**Published:** 2020-09-21

**Authors:** Julia Greenfield, Xiaoran Shang, Heng Luo, Yan Zhou, Sara B. Linden, Ryan D. Heselpoth, Petr G. Leiman, Daniel C. Nelson, Osnat Herzberg

**Affiliations:** 1 Institute for Bioscience and Biotechnology Research, University of Maryland, 9600 Gudelsky Dr, Rockville, MD 20850 USA; 2 Department of Chemistry and Biochemistry, University of Maryland, 0107 Chemistry Building, 8051 Regents Dr, College Park, MD 20742 USA; 3 Department of Biochemistry and Molecular Biology, Sealy Center for Structural Biology and Molecular Biophysics, University of Texas, Medical Branch, 301 University Blvd, Galveston, TX 77555 USA; 4 Department of Veterinary Medicine, University of Maryland, 8075 Greenmead Dr, College Park, MD 20740 USA

**Keywords:** Biochemistry, Structural biology

## Abstract

The genome of *Escherichia coli* O157:H7 bacteriophage vB_EcoM_CBA120 encodes four distinct tailspike proteins (TSPs). The four TSPs, TSP1-4, attach to the phage baseplate forming a branched structure. We report the 1.9 Å resolution crystal structure of TSP2 (ORF211), the TSP that confers phage specificity towards *E. coli* O157:H7. The structure shows that the N-terminal 168 residues involved in TSPs complex assembly are disordered in the absence of partner proteins. The ensuing head domain contains only the first of two fold modules seen in other phage vB_EcoM_CBA120 TSPs. The catalytic site resides in a cleft at the interface between adjacent trimer subunits, where Asp506, Glu568, and Asp571 are located in close proximity. Replacement of Asp506 and Asp571 for alanine residues abolishes enzyme activity, thus identifying the acid/base catalytic machinery. However, activity remains intact when Asp506 and Asp571 are mutated into asparagine residues. Analysis of additional site-directed mutants in the background of the D506N:D571N mutant suggests engagement of an alternative catalytic apparatus comprising Glu568 and Tyr623. Finally, we demonstrate the catalytic role of two interacting glutamate residues of TSP1, located in a cleft between two trimer subunits, Glu456 and Glu483, underscoring the diversity of the catalytic apparatus employed by phage vB_EcoM_CBA120 TSPs.

## Introduction

*Escherichia coli* phage vB_EcoM_CBA120 (CBA120) is an architypical species of the newly created *Kuttervirus* genus in the *Ackermannviridae* family^1^, which was ratified by the International Committee on Taxonomy of Viruses (https://talk.ictvonline.org). The *Kuttervirus* are a closely related genus of contractile tailed phages that infect *Escherichia* and *Salmonella* species and often display multiple tailspike proteins (TSPs) and tail fibers on the phage particle. These receptor-binding proteins enable individual phage to recognize and infect a range of bacteria. Indeed, infection of multiple bacterial species has been described for the *Kuttervirus* phage Sfp10^2^. Likewise, CBA120 with its four TSPs (TSP1-TSP4, encoded by ORFs 210–213, respectively) infects enterohemorrhagic *E. coli* O157:H7^3–5^, *E. coli* O77, O78, and *Salmonella enterica* serovar Minnesota^6^. CBA120 lytic activity towards *E. coli* O157:H7 is of particular interest because it may be developed as a potential new bio-indicator or biocontrol agent.

**Figure 1:**
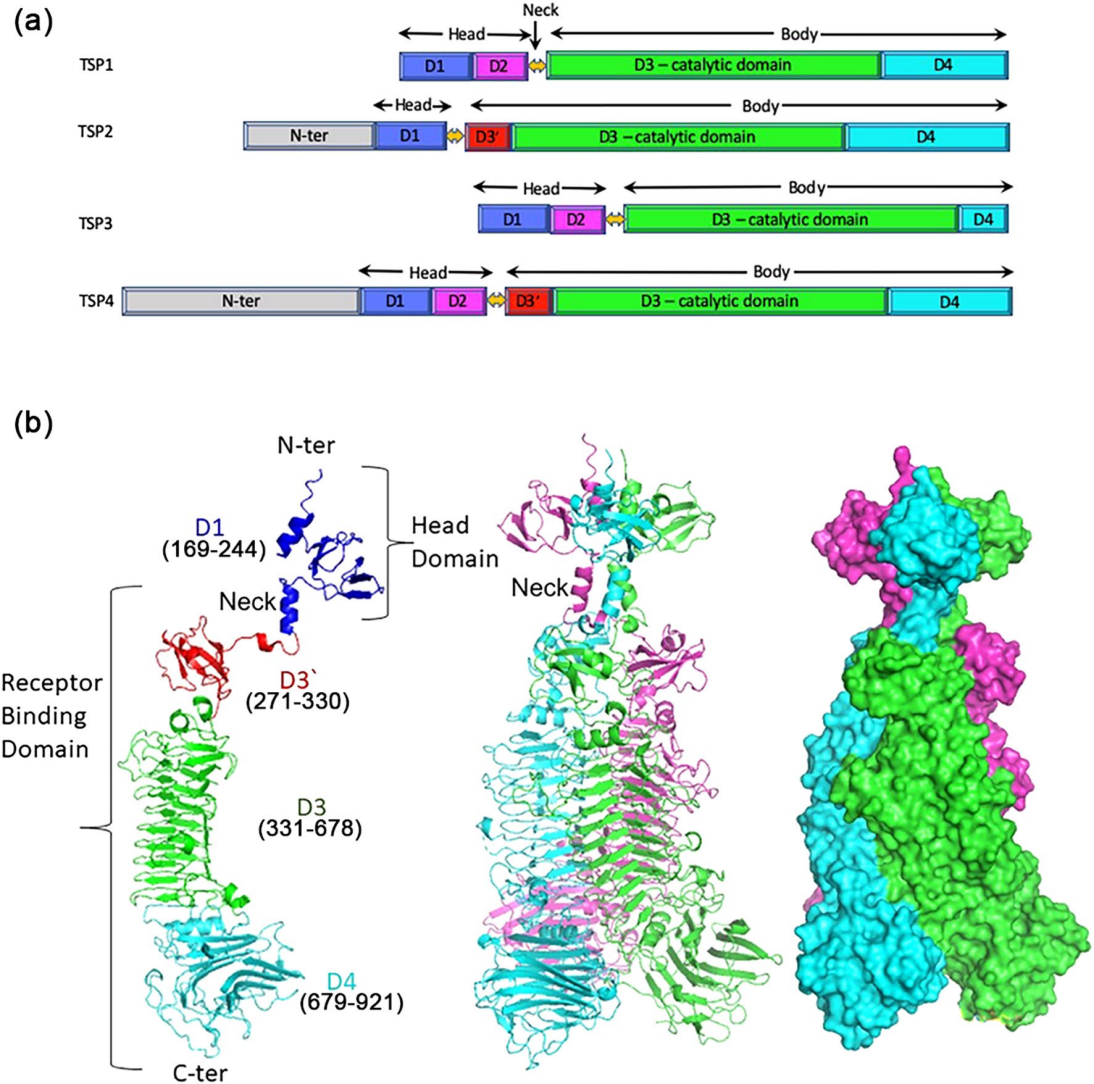
Domain structures of CBA120 TSPs and overall structure of TSP2. (**a**) Schematic diagram of TSP1-4 domain arrangements. The N-terminal regions (N-ter) of TSP2 and TSP4 contain 2 and 4 domains, respectively. These domains are involved in protein–protein interactions and are not depicted here because the TSP2 N-ter region (residues 1–168) is structurally disordered. (**b**) A cartoon representation of the monomer (left), the trimer (middle), and the trimer surface representation of TSP2 (right). Each molecule of the trimer assembly is colored differently. The figure was generated using the computer program PyMol v1.8.0.2 (Schrödinger, LLC, https://www.pymol.org).

TSPs serve as receptor binding proteins that allow for the initial reversible contact between the phage and the bacterial cell. After binding to the bacterial lipopolysaccharide (LPS), TSPs exhibit lyase, glycosidase or esterase activity directed toward the O-antigen part of the LPS that eventually results in irreversible binding of the phage particle to its bacterial target^7,8^. For consistency, the terminology used to describe the architecture of TSP1 and TSP3 structures will be used here^9,10^. TSPs form homotrimers, which in the case of TSP1 and TSP3 are broadly divided into two domains. The N-terminal domain, termed here the head domain, binds to partner TSPs (TSP2 or TSP4), and the C-terminal “body” domain contains the polysaccharide receptor binding/catalytic site (Fig. 1a). TSP2 and TSP4 also contain head and body domains^6^. However, preceding the head domains are N-terminal regions of undetermined structures, approximately 170- and 340-residues long, respectively, which contain the binding sites for TSP1 and TSP3 head domains (Fig. 1a). Together, the assembly of the four TSPs form a branched structure that attaches to the CBA120 baseplate^6^. Analogous assembly domains were reported for phage G7C and for adaptor protein^11,12^.

The head region of each trimer subunit of TSP1, TSP3, and TSP4 comprises approximately 170-residue long polypeptide that folds into two independent structural units, designated here D1 and D2 (corresponding to TD1 and TD2 in the terminology used by Plattner et al.^6^). The crystal structure of TSP2 lacking both the assembly and head domains with and without a bound tetrasaccharide derived from the *E. coli* O157:H7 LPS was reported^6^. Within the body region, each trimer subunit of TSP1 and TSP3 contains two different fold modules, designated D3 and D4 (Fig. 1a). D3 carries the polysaccharide binding/catalytic activity and adapts a 3-faced β-helix fold shared by all TSPs that cleave glyosidic bonds and is referred to as the catalytic domain. The ladder length of the β-helix varies in each TSP as are the sizes and conformations of solvent-exposed connecting loops between ladder steps. The C-terminal domains, D4, form β-structures of diverse classes, and their roles are not clear. A three α-helix bundle, termed “neck”, connects the head and body domains of TSP1 and TSP3, as first seen in the crystal structure of a D3-D4 fragment of phage P22 TSP, which included the neck but not the head domain, and confirmed later in the structure of the intact protein^13,14^. TSP2 and TSP4 also contain necks and D3-D4 domains^6^. However, an additional fold module is inserted between the neck’s α-helix and D3 of both TSP2 and TSP4, which is designated here D3′ (Fig. 1a).

The four CBA120 TSP structures revealed that the glycosidase catalytic machinery, which most commonly involve the carboxylate groups of pairs of aspartic or glutamic amino acids, are likely to be located along the interface between adjacent trimer subunits. Interestingly, the spatial arrangement of the carboxylate groups and the environment that supports catalysis differs in each TSP. The identity of the catalytic residues of TSP3 was confirmed by site-directed mutagenesis (Glu362 and Asp426, each on a different subunit)^10^. For TSP2, the tetrasaccharide building block of the *E. coli* O157:H7 O-antigen bound close to an inter-subunit carboxylate pair (Asp506 and Asp571 located on adjacent subunits), implicating these amino acids in the catalytic machinery. However, problems with the production of soluble site-directed mutants prevented direct experimental validation of the identity of the catalytic residues^6^.

Herein, we describe the crystal structure of full-length TSP2, which reveals a small head domain formed by a D1 fold module, lacks the D2 fold module, and its N-terminal 168 amino acids corresponding to the interaction region of TSP4 are structurally disordered. We identify the catalytic residues by analyzing the properties of site-directed mutants and employ an assay that shows that in addition to TSP2, which previously was reported to degrade the O-antigen of *E. coli* O157:H7^6^, TSP1, TSP2, and TSP3 act on non-O-antigen components, which may include outer core polysaccharides of the LPS or non-LPS surface polysaccharide yet to be identified.

## Results and discussion

### Protein oligomerization state and stability

Analytical size-exclusion chromatography of purified TSP2 revealed a single homogeneous peak at ~ 470 kDa, suggesting that the protein forms oligomers in solution (the calculated monomer molecular weight is 99,054 Da for the 6X-His tagged protein). The accuracy of the method is insufficient to determine the exact oligomeric state, perhaps because of the elongated nature of the three-dimensional structure. The crystal structure reported here clearly shows that similar to all other TSPs with known structures^15^, TSP2 forms a trimer.

TSP2 loss of β-sheet content as a function of temperature was monitored by circular dichroism (CD) spectroscopy. When heated from 20 to 95 °C, the protein unfolded cooperatively with a melting temperature (T_m_) of 82.8 °C (data not shown). Similar high thermal stability of tailspikes, thought to be required to withstand harsh environmental conditions, has been reported in the literature for TSP1 of phage CBA120 (T_m_ = 80.7 °C), the TSP of phage P22 (T_m_ = 88.4 °C) and the TSP of phage HK620 (T_m_ = 80 °C)^9,16,17^. However, the thermal stability of TSP3 from phage CBA120 is considerably lower (T_m_ = 61.8 °C)^10^.

### TSP assays

**Figure 2:**
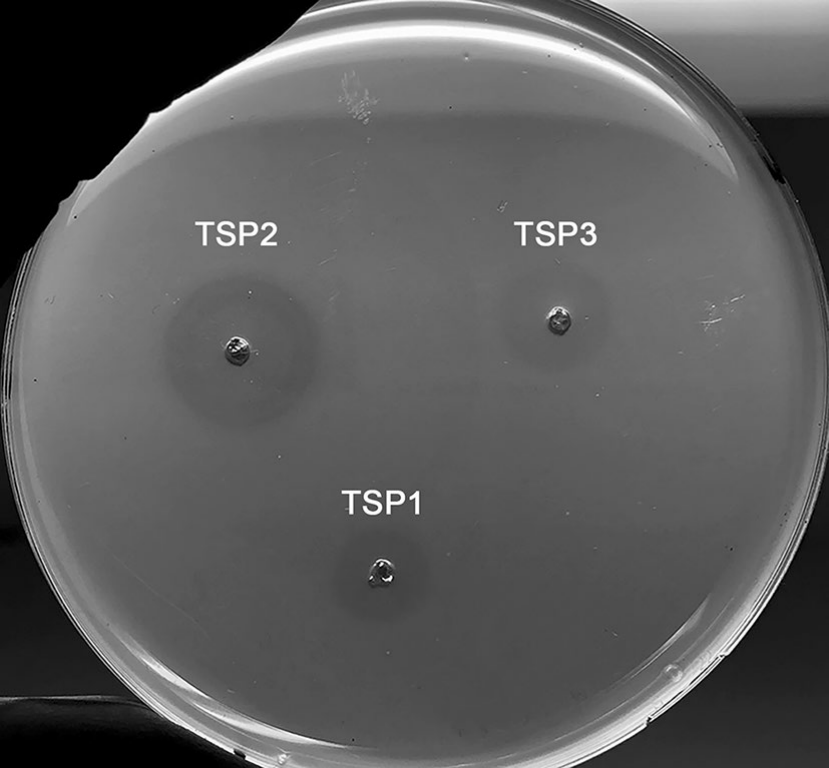
Halo assay of TSP1, TSP2, and TSP3. *E. coli* strain ATCC 700,728 was embedded in agarose. Wells (3 mm) were cut out of the agarose and loaded with 10 µL (6 mg/mL) of TSPs. Following overnight incubation at 37 °C, each TSP produced a halo, which is indicative of glycosidase activity.

TSPs do not lyse bacterial cells. Nevertheless, by cleaving or modifying surface polysaccharides, they reduce the thickness of bacterial outer layer embedded in an agar dish, and this reduction can be viewed as a halo of less opacity. We previously used the halo assay to follow the glycosidase activity of TSP3^10^. This assay enabled probing active site residues of TSP3 by site-directed mutagenesis. Replacing Glu362 and Asp426 with Gln and Asn, respectively, resulted in a protein incapable of generating a halo, thus identifying the catalytic machinery. We noted that the assay does not determine the identity of the specific polysaccharide that is being cleaved, nevertheless it does allow for semi-quantitative demonstration and comparison of activity. We now show that the glycosidase activity of TSP1 and TSP2 can also be monitored using the halo assay. Figure 2 shows that wild-type TSP1, TSP2, and TSP3 all generate halos on an *E. coli* O157:H7 agar plate. The halo produced by TSP2 is the largest, perhaps because in addition to the surface polysaccharide(s) cleaved by TSP1 and TSP3, TSP2 also specifically digests the O157 antigen^6^.

The halo data support the conclusion that CBA120 TSPs can cleave non -antigen oligosaccharide on the surface of *E. coli* O157:H7. To demonstrate non-O-antigen glycosidase activity of the TSPs, we examined knockout mutants of *E. coli* O157:H7, which removed either *galU* or *galETKM* of the *gal* operon encoding enzymes that catalyze the synthesis of *N*-acetyl-d-galactose^18^. These *E. coli* O157 mutants are devoid of O-antigen because they lack the required N-acetyl-D-galactose component. Similar to TSP3^10^, both TSP2 and TSP1 produced halos on these *E. coli* O157:H7 mutants (data not shown), suggesting that all three TSPs are capable of cleaving non-O-antigen moieties of the LPS or even an entirely different surface polysaccharide, thus altering the opacity of the cell matrix.

**Figure 3:**
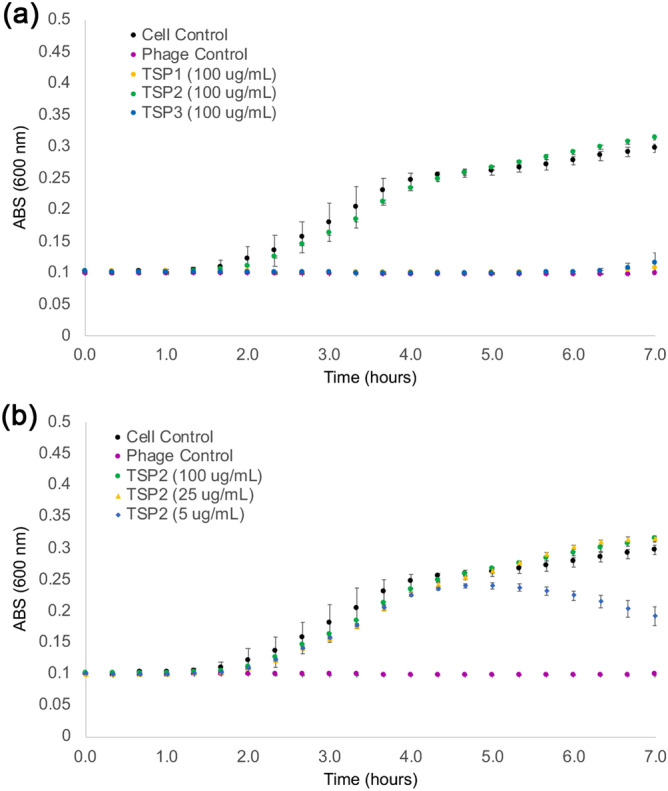
Phage infection assays. Phage CBA120 infection of *E. coli* strain ATCC 700,728 was followed by spectrophotometry at 600 nm as detailed in the methods section. The mean values of technical triplicate and the standard deviations are shown (**a**) Phage infection in the presence of various TSPs. The ability of CBA120 to infect *E. coli* O157:H7 treated with TSP1 (yellow circles), TSP2 (green circles), or TSP3 (blue circles) at 100 μg/mL is shown together with controls consisting of bacterial cells alone (black circles) and non-TSP treated bacterial cells incubated with phage (magenta circles). Only treatment with TSP2 prevented phage infection and subsequent bacterial death. (**b**) Phage CBA120 infection dependence on TSP2 concentration. Controls are the same as in (**a**). TSP2 at 25 and 100 μg/mL prevented killing by the phage but 5 μg/mL inhibited phage killing only for ~ 4.5 h.

A turbidity assay was used to identify which TSP is responsible for the specificity towards the *E. coli* O157:H7 LPS, the polysaccharide that is required for phage CBA120 infectivity. In this assay, varying concentrations of TSPs (5–100 µg/mL) were incubated at 37 °C together with the phage and bacterial culture, and bacterial growth was monitored by measuring the optical density at 600 nm. It was reported earlier that under anaerobic conditions, the bacteria grew for 90 min after phage inoculation, and then growth declined due to lysis by phage CBA120^4^. The specificity assay shows which TSP competes with the phage binding to the bacterial receptor. Controls to establish baseline curves for normal bacterial growth and bacterial killing by phage CBA120 showed that bacterial growth was halted for ~ 6 h, and then the bacteria recovered and proliferated at the same rate as the culture without the added phage. In addition, turbidity assays with the *E. coli* O157:H7 *gal* mutants, TEA023, TEA026, and TEA028, which lack the O-antigen^18^, confirmed that phage CBA120 was unable to infect and lyse LPS-defective *E. coli* O157:H7. Figure 3a shows that of the three TSPs tested, only TSP2 impaired phage infection, and Fig. 3b shows TSP2′s concentration-dependent competition with phage CBA120 for the LPS binding sites. The highest concentration of TSP2, 100 µg/mL, abolished phage CBA120 infectivity, and this culture growth curve is similar to that of the cell controls without phage. These experiments agreed with results obtained recently by Plattner et al.^6^ that demonstrated that TSP2 competitively inhibited CBA120 proliferation in a soft agar assay and that TSP2 specifically degraded the *E. coli* O157:H7 O-antigen whereas TSP1 or TSP3 did not.

### Overall crystal structure

A TSP2 crystal suitable for data collection was obtained only once, and later attempts to reproduce the crystals failed. Consequently, the TSP2 structure could be determined by molecular replacement only after the structure of TSP2 whose assembly and head domains have been truncated was determined^6^. The TSP2 structure was refined at 1.9 Å resolution (Table 1). The crystal asymmetric unit contains two homotrimers, providing two independent views of the biological trimer. For both trimers, 168 N-terminal amino acids have no associated electron density; thus, these were not modeled. These residues comprise the region that interacts with other TSPs and exhibit remote sequence homology to proteins of known structure (phage T4 gp10) that can be detected only by Hidden Markov model methods^6^. As the crystals could not be reproduced, it is unknown whether this N-terminal region was cleaved by a contaminating protease or whether it is structurally disordered. Nevertheless, the electron density map contains large solvent channels in the vicinity of the visible N-termini, sufficient to accommodate the missing 168 residues. The C-terminal 6xHis affinity tag is by and large disordered, with only a few histidine residues seen in the electron density map.

**Table 1:** Statistics of data collection and refinement of CBA120 TSP2.

**Data collection**	
Wavelength (Å)	1.0332
Resolution (Å)^a^	48.36–1.85 (1.88–1.85)
Space group	P 2_1_2_1_2_1_
Unit cell dimension (Å, °)	a = 83.0, b = 259.0, c = 269.7
No. of molecules in the asymmetric unit	6 (two trimers)
No. of unique reflections^a^	491,364 (24,214)
Multiplicity^a^	5.0 (5.0)
Completeness (%)^a^	99.8 (100.0)
Mean I/σ(I)^a,b^	7.9 (0.9)
*R*_*merge*_^a^	0.131(2.175)
**Refinement**	
Resolution (Å)^a^	48.36–1.9 (1.95–1.90)
Total no. of reflections^a^	430,672 (31,614)
*R*_*work*_/*R*_*free*_^a,b^	0.168 (0.319)/0.188 (0.316)
No. protein residues	4,533
Ligands	117
Solvent	3,887
RMSD from ideal geometry bonds length (Å)/bond angles (°)	0.011/1.74
Ramachandran plot: favored/allowed/outliers (%)	95.13/4.62/0.24

^a^The values in parentheses are for the highest resolution shell.

^b^*R*_*merge*_ = *∑*_*hkl*_*∑*_*j*_*|I*_*j*_*(hkl)* *−* < *I(hkl)* >*|*/*∑*_*hkl*_* ∑*_*j*_*I*_*j*_*(hkl).*

*R*_*work*_ = *∑*_*hkl*_ |*F*_*o*_ *−* *F*_*c*_|/*∑*_*hkl*_* F*_*o*_, where *F*_*o*_ and *F*_*c*_ are the observed and calculated structure factors, respectively.

*R*_*free*_ is computed from 5% of randomly selected reflections and omitted from the refinement.

**Figure 4:**
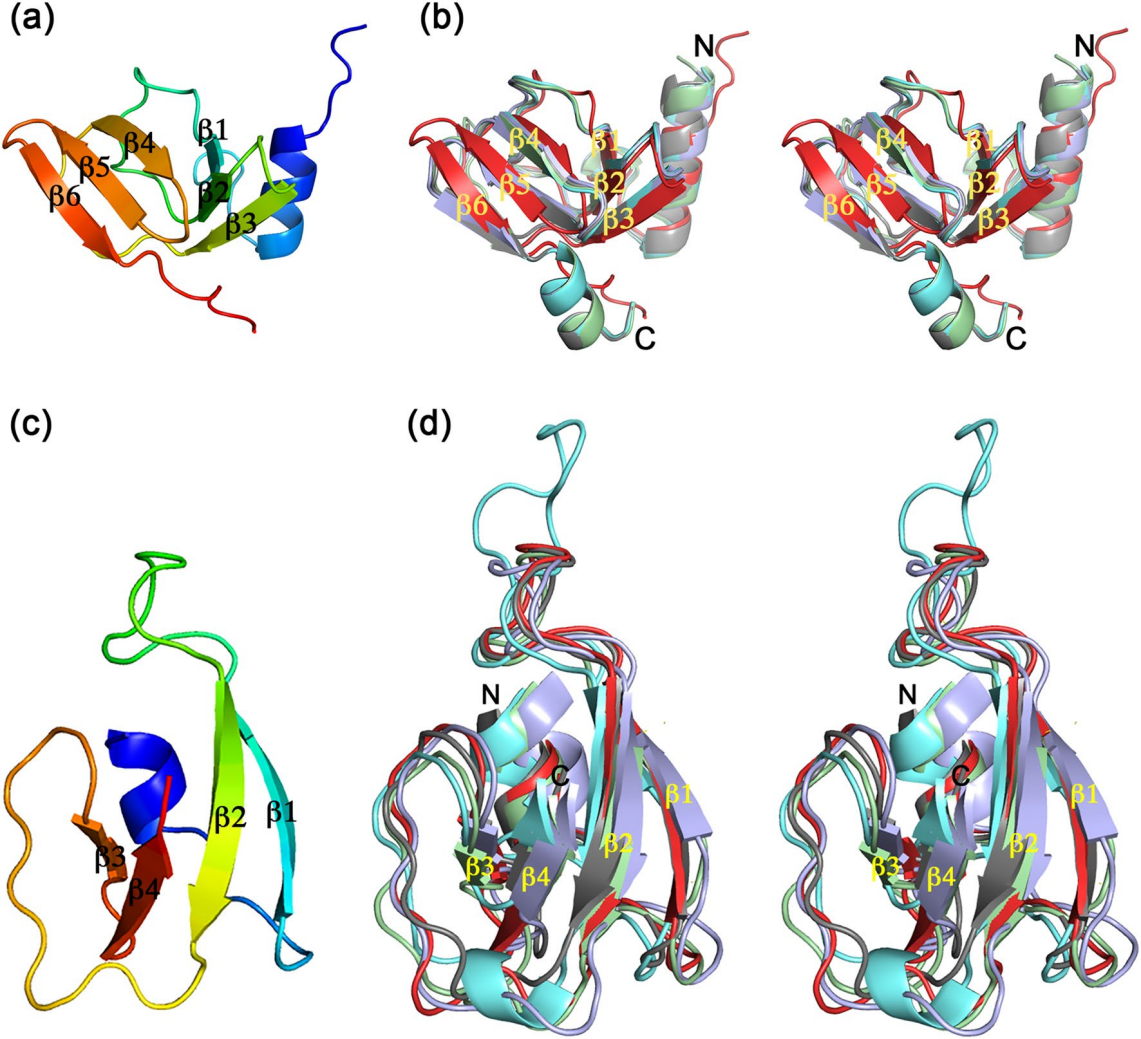
TSP2 D1 and D3′ folds. **(a**) Cartoon representation of D1 with rainbow coloring from the N-terminus (blue) to the C-terminus (red). (**b**) Stereoscopic view of superimposed D1 fold modules of TSP2 (red; residues q66-247), TSP4 (gray; residues 340–421 PDB entry **5W6H**)), TSP1 (green; residues 12–96, PDB entry code **4OJ5**), TSP3 (light blue; residues 12–95, PDB entry **6NW9**), and TSP gp63.1 from *E. coli* 4 s G7C phage (cyan; residues 13–96, PDB entry **4QNL**). (**c**) Cartoon representation of the D3′ with rainbow coloring from the N-terminus (blue) to the C-terminus (red). (**d**) Stereoscopic view of superimposed D3′ of TSP2 (red; residues 270–328), TSP4 (gray; residues 503–557 PDB entry 5W6H), TSP gp49 from *A. baumannii* phage Fri1 (green; residues 209–269, PDB entry **6C72**), TSP from *A. baumannii* phage AM24 (light blue; residues 37–96, PDB entry **5W5P**), TSP gp42 from *A. baumannii* phage vb_AbaP_AS12 (cyan; residues 23–82, PDB entry **6EU4**). The figure was generated using the computer program PyMol v1.8.0.2 (Schrödinger, LLC, https://www.pymol.org).

The TSP2 trimer assembles into a 160 Å elongated structure with a shape common to other TSP glycosidases, distinguished by having a head-like region, neck, and an elongated body (Fig. 1b). The surface area of each trimer subunit is 29,000 Å^2^ as calculated by the program PISA implemented at the European Bioinformatics Institute (https://www.ebi.ac.uk/pdbe/prot_int/pistart.html)^19^. The association into trimers buries over 22,000 Å^2^ surface area; i.e. > 7,000 Å^2^ per subunit, which is ~ 25% of the total subunit surface area. The structured region comprising amino acids 169–921 can be broadly divided into the two canonical tailspike domains; the N-terminal head domain (residues 169–244, or ~ 8% of the entire 921-residue TSP2 polypeptide chain) and the C-terminal body domain (residues 256–921, or ~ 72% of the entire TSP2 polypeptide chain), which are connected by a 12-residue α-helical “neck” (Fig. 1b). TSP2 head domain is strikingly smaller than the head domains of TSP1, TSP3, and TSP4; whereas each of the latter TSP head domains contains two adjoining fold modules, termed previously D1 and D2^9,10^, TSP2 head domain contains only the D1 unit and lacks the D2 unit (Figs. 1b and 4a). The TSP2 D1 exhibits the same fold as that of TSP1, TSP3, TSP4, and gp63.1 of phage G7C (Fig. 4b). Interestingly, gp63.1 of phage G7C is a deacylase rather than glycosidase and accordingly, the fold of its catalytic domain differs from that of the TSP glycosidases^12^, which exemplifies repeated utilization of the same structural modules during evolution for performing similar functions. Using the computer program PyMol (Schrödinger, LLC), pairwise superposition of TSP2 D1 with those of TSP1, TSP3, and TSP4 and gp63.1 resulted in root mean squares deviations (RMSD) in the range of 1.1–1.8 Å for 50–55 common Cα atoms. In contrast, TSP1, TSP3, TSP4 and gp63.1 D1 fold modules resemble one another more closely, with RMSD in the range of 0.2–0.4 for 70–73 common Cα atoms. The structural divergence of TSP2 D1 may be attributed to the lower sequence identity it shares with the D1 fold modules of TSP1, TSP3, TSP4, and gp63.1 (~ 35%), in contrast to the much higher sequence identity (~ 70%) between the latter four D1 fold modules.

**Figure 5:**
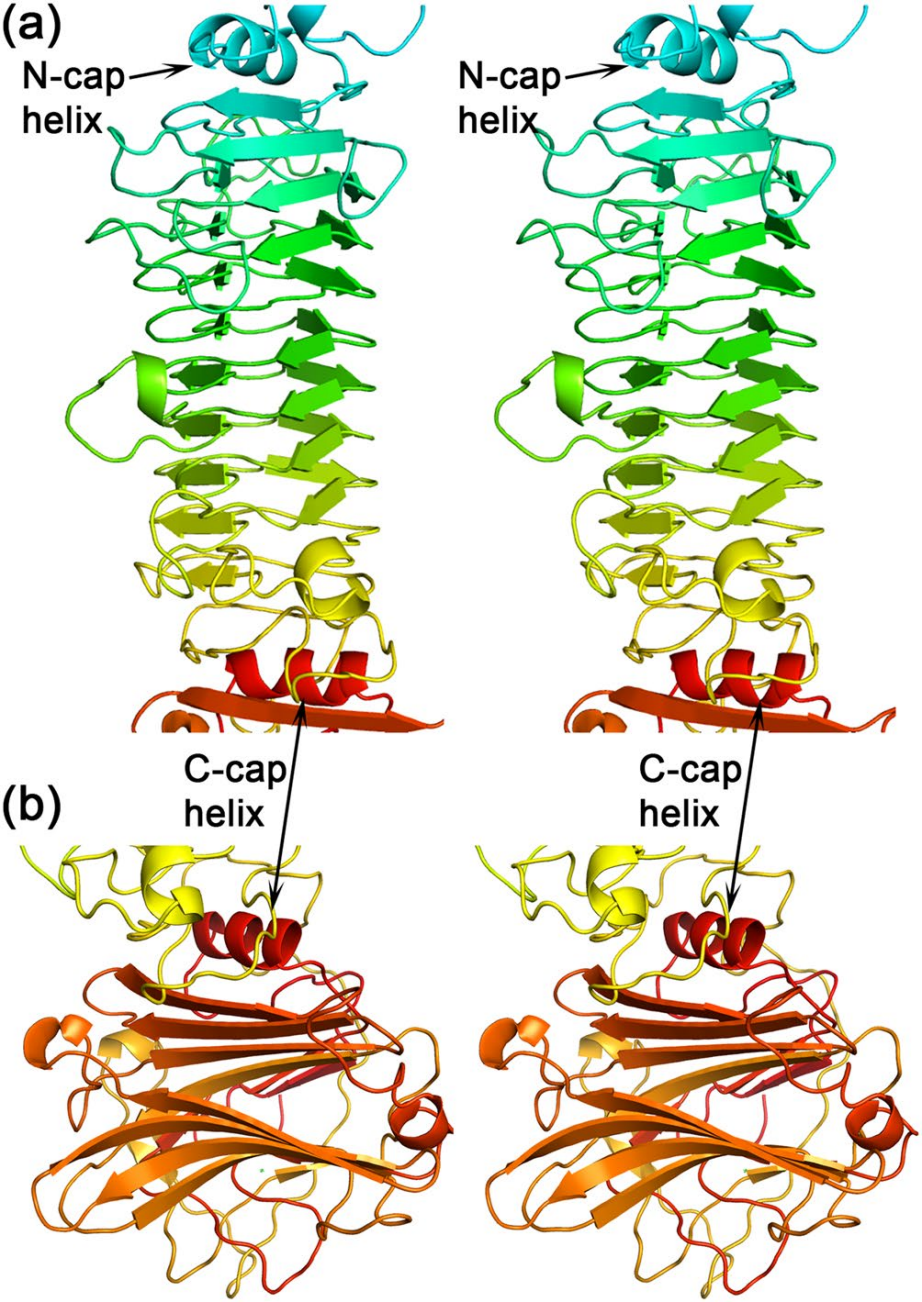
Stereoscopic representation of the TSP2 D3 and D4 folds. The polypeptide chain is colored with a rainbow color scheme beginning with blue at the N-terminus and ending in red at the C-terminus of the entire molecule. (**a**) The D3 β-helix, indicating the two capping α-helices. (**b**) The D4 β-sandwich. The D4 α-helix is close to the C-terminus of the polypeptide chain and caps the D3 β-helix C-terminus, a feature unique to TSP2. The figure was generated using the computer program PyMol v1.8.0.2 (Schrödinger, LLC, https://www.pymol.org).

The C-terminal domains of TSP glycosidases contain two adjoining fold modules, which we termed previously D3 and D4^9,10^. The enzymatic machinery resides on D3. The canonical D3 fold begins with a capping α-helix and an ensuing right-handed three-faced β-helix fold (Fig. 5a). The β-helix of TSP2 has eleven complete turns, although some β-strands on each face are distorted and do not form all the hydrogen bonds expected of a perfect β-sheet. The first β-helical face ensuing the helical cap contains an additional β-strand, i.e. this β-sheet ladder has 12 rungs.

The β-helix topology of D3 is conserved, albeit with considerable diversity in length and loop conformations. In contrast, numerous D4 β folds have been observed in crystal structures. The TSP2 D4 (as well as that of TSP4^6^) adopts a twisted antiparallel β-sandwich fold of complex topology, with six β-strands per β-sheet (albeit, 2 β-strands do not form optimal hydrogen bond interactions; Figs. 1b and 5b). The D4 fold modules of TSP2 and TSP4 are much larger than their TSP1 and TSP3 counterparts, and as seen in Fig. 1b, they splay apart from the D3 β-helix. As noted previously, D4 exhibits structural similarity to several carbohydrate binding proteins^6^. In TSP2 (but not TSP4), an α-helix preceding the D4 C-terminal β-strand is placed between the D3 and D4. Consequently, both N- and C-termini of the D3 are capped by α-helices (Fig. 5a,b). The entire D4 α-helix is buried in the protein core, an unusual structural feature.

Unlike TSP1 and TSP3, both TSP2 and TSP4 contain an additional fold module preceding D3 (designated D3′), which adopts an open face β-sandwich fold comprising an antiparallel 4-stranded β-sheet with β-strand topology 1 2 4 3 warped around a 2-turn α-helix (Fig. 4c). This compact fold module is inserted between the neck α-helix and the D3 capping α-helix (Fig. 1b). In contrast, the corresponding regions of TSP1 and TSP3 contain non-globular meandering chains. Dali^20^ structural comparison of the 59-residue D3′ fold revealed three more structures with the same fold, all observed in TSPs of phages that infect *Acinetobacter baumannii* and all preceding the D3 β-helix (Fig. 4d): TSP gp49 from phage Fri1 (PDB entry code **6C72**, Z = 10.1, RMSD for 58 aligned Cα atoms = 1.0 Å, amino acid sequence identity = 31%); TSP from phage AM24 (PDB entry code **5W5P**, Z = 9.0, RMSD for all 59 Cα atoms = 1.3 Å, amino acid sequence identity = 37%); and TSP gp42 from phage vb_AbaP_AS12 (PDB entry code **6EU4**, Z = 7.1, RMSD for 51 aligned Cα atoms = 1.5 Å, amino acid sequence identity = 24%). The D3′ fold topology has been observed only in TSPs even though the open face β-sandwich is a common fold.

### Intra and inter molecular interactions

**Figure 6:**
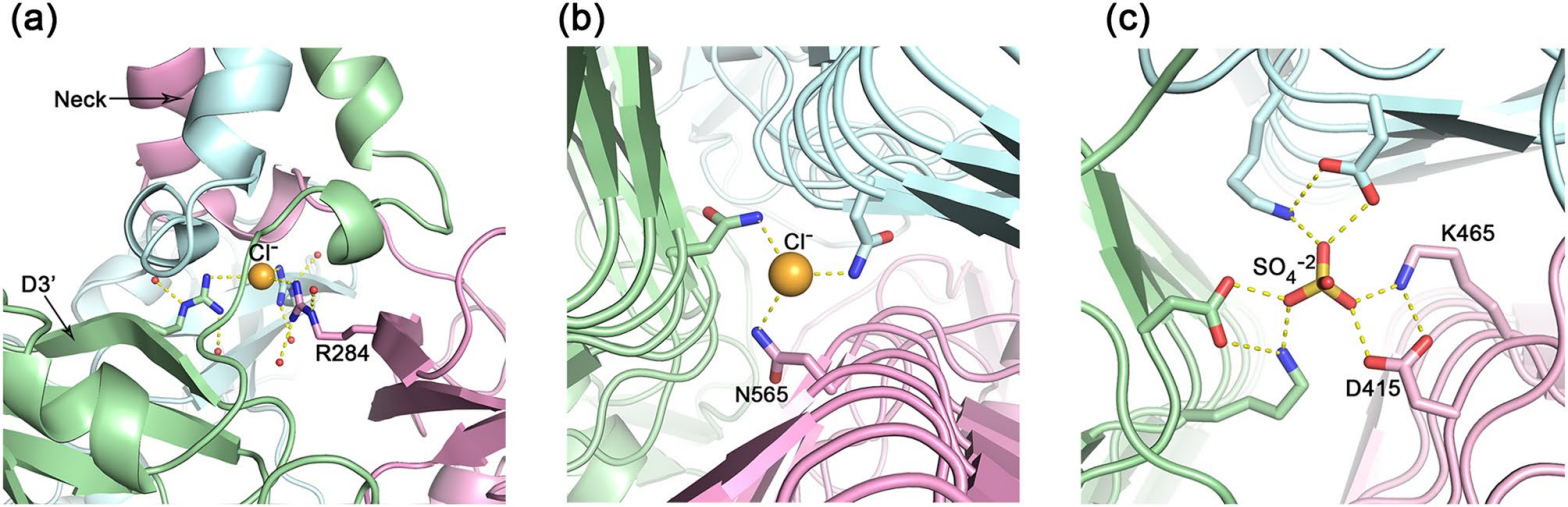
Anions located along the TSP2 threefold axis support trimer oligomerization. (**a**) A chloride located between the neck and the D3′ domain forms charge-charge interactions with three guanidinium groups of Arg284. (**b**) A chloride located along the intermolecular D3 β-helices axis interacts with three side chain amide groups of Asn565. (**c**) A sulfate located along the intermolecular D3 β-helices axis form charge-charge interaction with the carboxyl groups of Asp415 and the amino groups of Lys465. The figure was generated using the computer program PyMol v1.8.0.2 (Schrödinger, LLC, https://www.pymol.org).

The cores of each TSP2 trimer subunits are packed with hydrophobic amino acids, as common in protein structures. TSP2 trimer association is mediated by the N-terminal α-helix of D1, the neck α-helix, and across neighboring D3 β-helices, whereas the D3′ and D4 units do not contribute much to the trimer association. The internal trimer channel along the trigonal axis is lined primarily with hydrophilic and charged amino acid side chains. Solvent molecules bind in the channel to support this arrangement, including anions and water molecules. Three anions were assigned in each trimer channel, two chlorides and a sulfate (Fig. 6). One chloride binds just underneath the neck at the top of the D3′. It forms charge-charge interactions with the three guanidinium groups of Arg284 on the trimer’s three D3′ units (Fig. 6a). The second chloride interacts with three Asn565 side chains on the eighth rung of the β-helix (Fig. 6b). This chloride is located underneath the active site described below, contributing to the integrity of the catalytic machinery. The assignment of chlorides was made based on the coordination distance (3.3–3.5 Å) and the electron density that could not be fully accounted by water molecules. The sulfate forms ionic interactions with Asp415 and Lys465 located on the third and fourth rungs of the β-helix, respectively, at the inner corner of the β-helix triangular cross section (Fig. 6c). While physiological solution contains chlorides, the sulfate was included in the crystallization solution but is more likely to be replaced by a phosphate under physiological conditions.

### Active site and catalytic machinery of TSP2

The glycosidase active sites of TSPs are always located within the D3 β-helix, but the exact location varies in each protein. Both intramolecular and intermolecular active site locations have been reported^14,16,21,22^. The common catalytic machinery of all glycosidase TSPs studied to date comprises two nearby carboxyl groups of Asp/Glu residues, consistent with an acid/base mechanism^23^. Based on the structure of TSP3, we proposed an intermolecular active site ^10^. A halo assay testing TSP3 site-directed mutants showed that the E362Q:D426N TSP3 generated no halo and therefore identified the catalytic residues as Glu362 and Asp426 located across the intermolecular crevice^10^.

**Figure 7:**
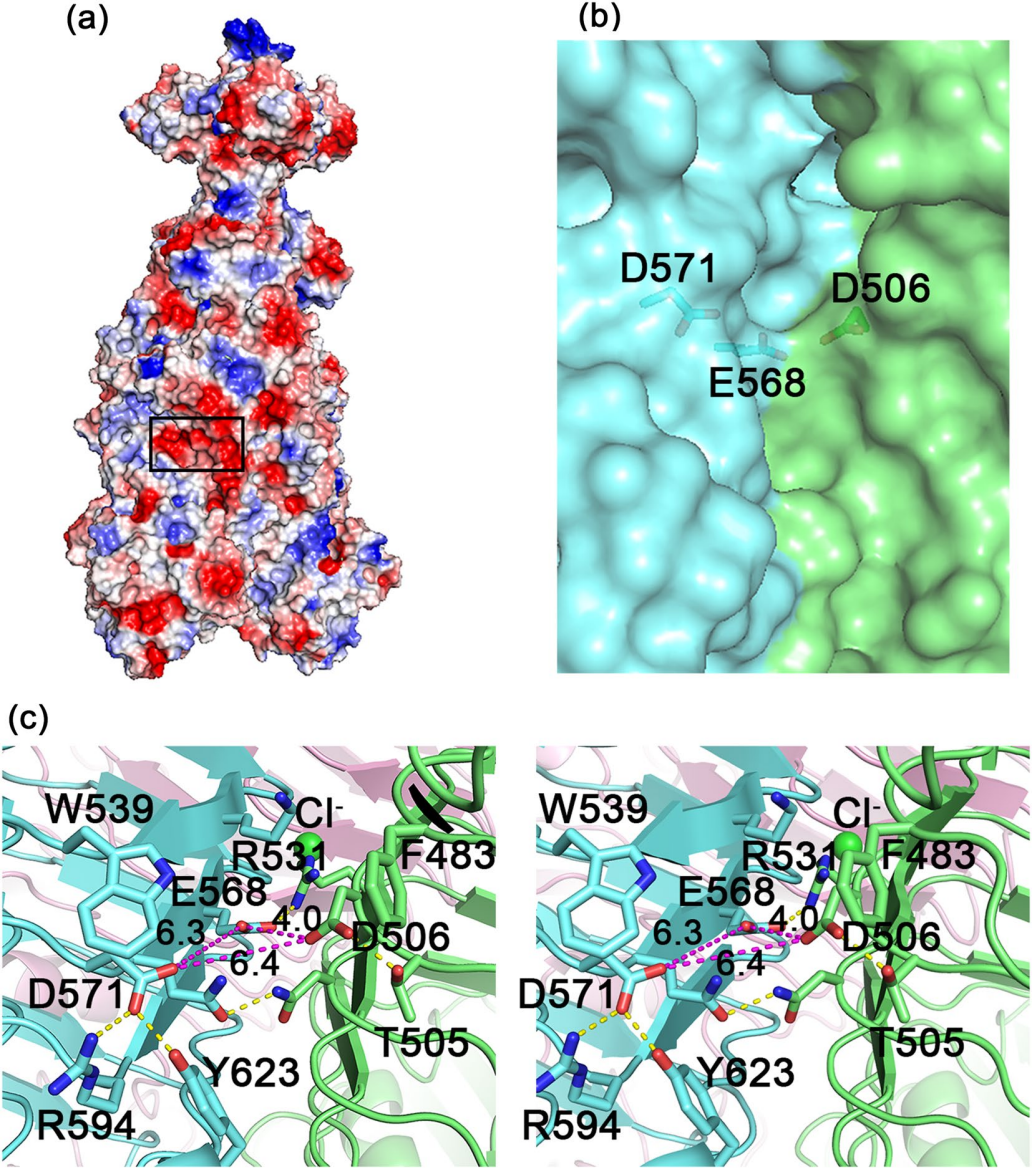
The active site of TSP2. (**a**) Surface vacuum electrostatics of the TSP2 trimer, indicating the location of the active site (rectangular black box). Negatively polar regions are colored red and positively polar region are colored blue. The overall polarity of the active site is negative. (**b**) The partially transparent molecular surface at the active site, highlighting key active site residues. As can be seen, the interface between the green and cyan molecules forms a cleft. The trimer contains three such clefts at the three subunit interfaces. (**c**) Stereoscopic representation of active site residues. The distances between the three carboxyl groups of Asp506, Asp571, and Glu578, the candidate catalytic resides that were probed by site-directed mutagenesis, are shown in magenta dash lines. Key salt bridges and hydrogen bond interactions are shown in yellow dash lines. Aromatic groups may interact with the substrate pyranose rings. The chloride bound underneath the active site is shown as green sphere. The trimer subunits are colored differently. The figure was generated using the computer program PyMol v1.8.0.2 (Schrödinger, LLC, https://www.pymol.org).

For TSP2, the structure suggests that the active site is also located within an intermolecular cleft, with Asp506 and Asp571 positioned 6.4 Å apart, and oriented appropriately to form the acid/base catalytic machinery (Fig. 7). Moreover, a TSP2 crystal soaked with the *E. coli* O157:H7 substrate revealed a tetrasaccharide bound in the vicinity of Asp571 and Asp506^6^. However single residue mutants remain active and the double mutant was insoluble^6^. The environment of the proposed active site has negative electrostatic potential (Fig. 7a), is enriched with charged and polar residues that interact with the carboxyl groups (Fig. 7c), and similarly to other oligosaccharide binding proteins, contains aromatic residues that may stack against the substrate pyranose rings (Fig. 7c). Glu568 is positioned close to Asp506 and Asp571 carboxyl groups (4.0 Å and 6.3 Å, respectively), but is more occluded from solvent compared with the two aspartic acids. Unique to TSP2, one of the trimer chlorides [the one interacting with three Asn565 sides chains (Fig. 6b)] is located underneath the proposed active site (Fig. 7c), thus supporting the structural integrity of the site.

**Figure 8:**
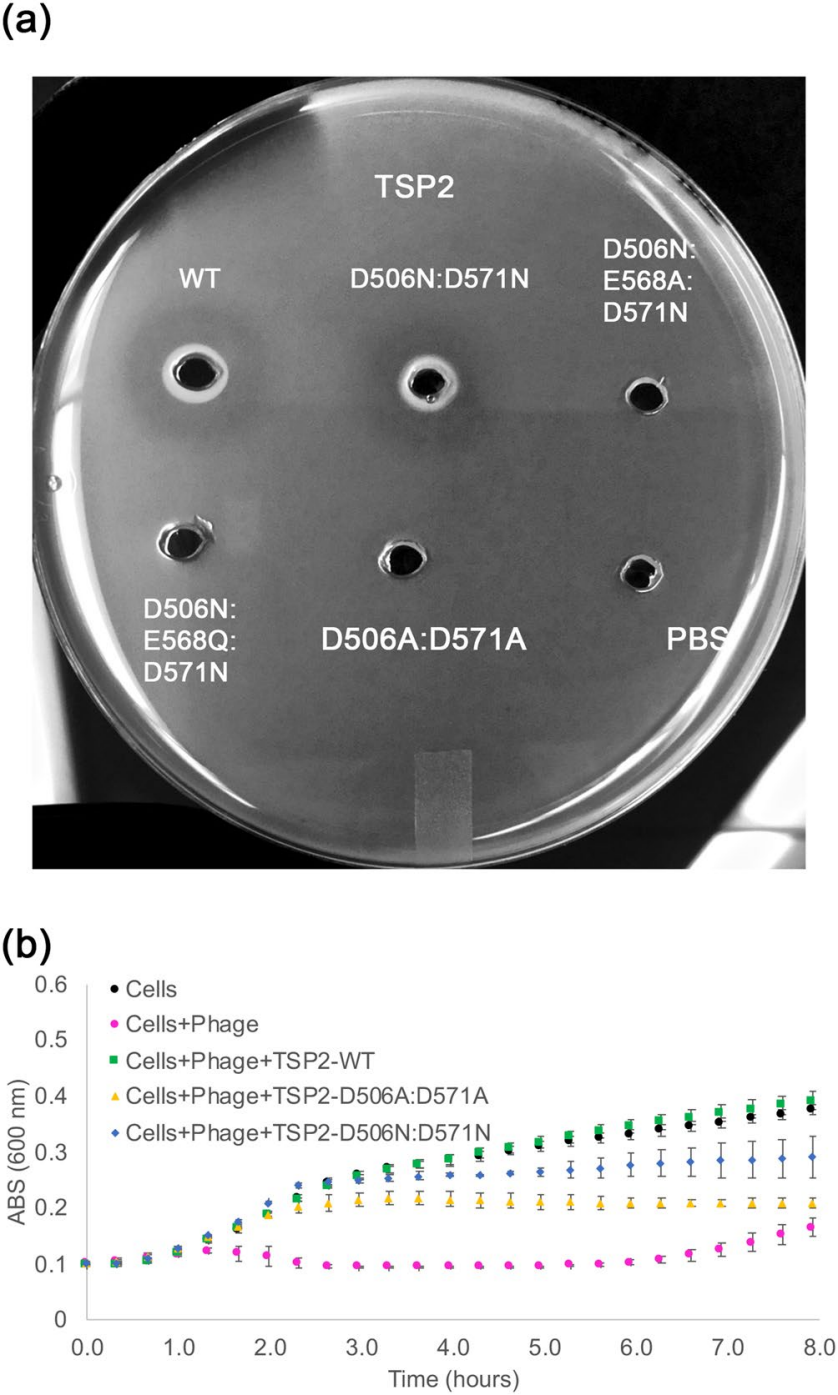
Assays to probe TSP2 active site mutants. Structure and mechanism-based analyses were used to identify the active site residues. (**a**) In the halo assay, *E. coli* strain ATCC 700,728 was embedded in agarose. Wells (3 mm) were cut out of the agarose and loaded with 10 μL (6 mg/mL) of wild-type TSP2 or active site TSP2 mutants, and incubated overnight at 37 °C to visualize glycosidase activity. The D506A:D571A TSP2 mutant was incapable of producing a halo, indicating glycosidase activity of TSP2 was abolished. Conversely, the D506:D571N TSP2 mutant retained enzymatic activity. However, introducing the E568A (i.e. D506N:E568A:D571N) or E568Q (i.e. D506N:D568Q:D571N) mutations to a D506N:D571N background inhibited TSP2 glycosidase activity, suggesting involvement of the Glu568 carboxyl group as part of an alternative catalytic machinery. Wild-type TSP2 and PBS only served as positive and negative controls for glycosidase activity, respectively. (**b**) In the bacterial infection assay, *E. coli* O157:H7 (ATCC 700728) culture was mixed with TSP variants as described in Methods. Absorbance measurements at 600 nm were made every 20 min. Bacterial growth is shown for *E. coli* incubated with phage following treatment with 100 μg/mL of either wild-type or TSP2 (green squares, D506:D571A TSP2 (yellow triangles), or D506N:D571N TSP2 (blue diamonds). Growth of bacterial cells alone (black circles) and non-TSP treated bacterial cells incubated with phage (magenta circles) served as controls. The mean values of technical triplicate and the standard deviations are shown.

We used the halo assay coupled with site-directed mutagenesis to probe potential TSP2 catalytic residues. All mutant proteins were produced and purified as soluble, stable, trimeric proteins. Figure 1 shows that wild type TSP2 generates a halo, indicative of polysaccharide degradation that changes the opacity of the bacterial culture on a Petri dish. Initially, two double mutants were prepared, D506A:D571A and D506N:D571N, and their effect on the development of a halo examined (Fig. 8a). Replacements of the two aspartic acids by alanine residues abolished the halo formation, thus confirming that Asp506 and Asp571 comprise the catalytic machinery. However, replacements by asparagine residues did not abolish halo formation despite the removal of the two carboxyl groups. Applying TSP2 and its active site mutants to CBA120 phage infection assays yielded consistent results (Fig. 8b). While both mutants impaired phage infection, the D506N:D571N TSP2 competed with phage binding better than the inactive D506A:D571A TSP2, which resulted in reduced bacterial lysis by the phage.

The difference between the TSP2 alanine and asparagine double mutants was surprising because the previous TSP3 activity mutants showed that replacement of the active site Asp426/Glu362 by Asn/Gln, respectively, abolished halo formation. Thus, two additional double mutants were prepared, D506N:D571A and D506A:D571N. Both of these mutants showed very faint halos, which was difficult to image and therefore are not shown here. Together, the data of these mutants confirmed the identity of the catalytic residues but the wild-type-like activity of the TSP2 asparagine double mutant was puzzling. We hypothesized that an alternative catalytic machinery arises in the presence of Asn506 and Asn571. The most likely catalytic residue candidate is Glu568 as it is positioned in the active site close to Asp506 and Asp571 (Fig. 7c). Indeed, replacements in the background of the two asparagine residues to produce the triple mutants D506N:E568A:D571N and D506N:E568Q:D571N resulted in proteins that did not produce halos (Fig. 8a), confirming that the carboxyl group of Glu568 can form part of an alternative catalytic machinery in the presence of the polar amide groups of Asn506 and Asn571.

The next question is which residue could partner with Glu568 to form an acid/base catalytic apparatus. We hypothesized that Tyr623 may have a reduced pKa when Asp571 is replaced by an asparagine because of its proximity to Arg594 (Fig. 7c). Tyr623 is located 8.9 Å away from the Glu568 carboxyl group, thus these two residues may form an alternative acid/base catalytic machinery. By comparison, although the active site of TSP3 also contains three carboxylate groups (Glu362, Asp 383 and Asp426) and an interacting Tyr (Y335), there is no nearby residues that can reduce the pKa of the tyrosine’s hydroxyl group^10^. Consequently, substitution of the catalytic Glu362 and Asp426 by glutamine and asparagine, respectively, abolishes the glycosidase activity of TSP3 and no alternative acid/base catalytic apparatus needs to be invoked. To test the role of Tyr623, the TSP2 D506N:D571N:Y623A mutant was purified as a soluble protein. However, the presence or absence of a halo was hindered by white aggregates, perhaps because protein binding caused clumping (data not shown).

### Identification of the TSP1 catalytic residues

**Figure 9:**
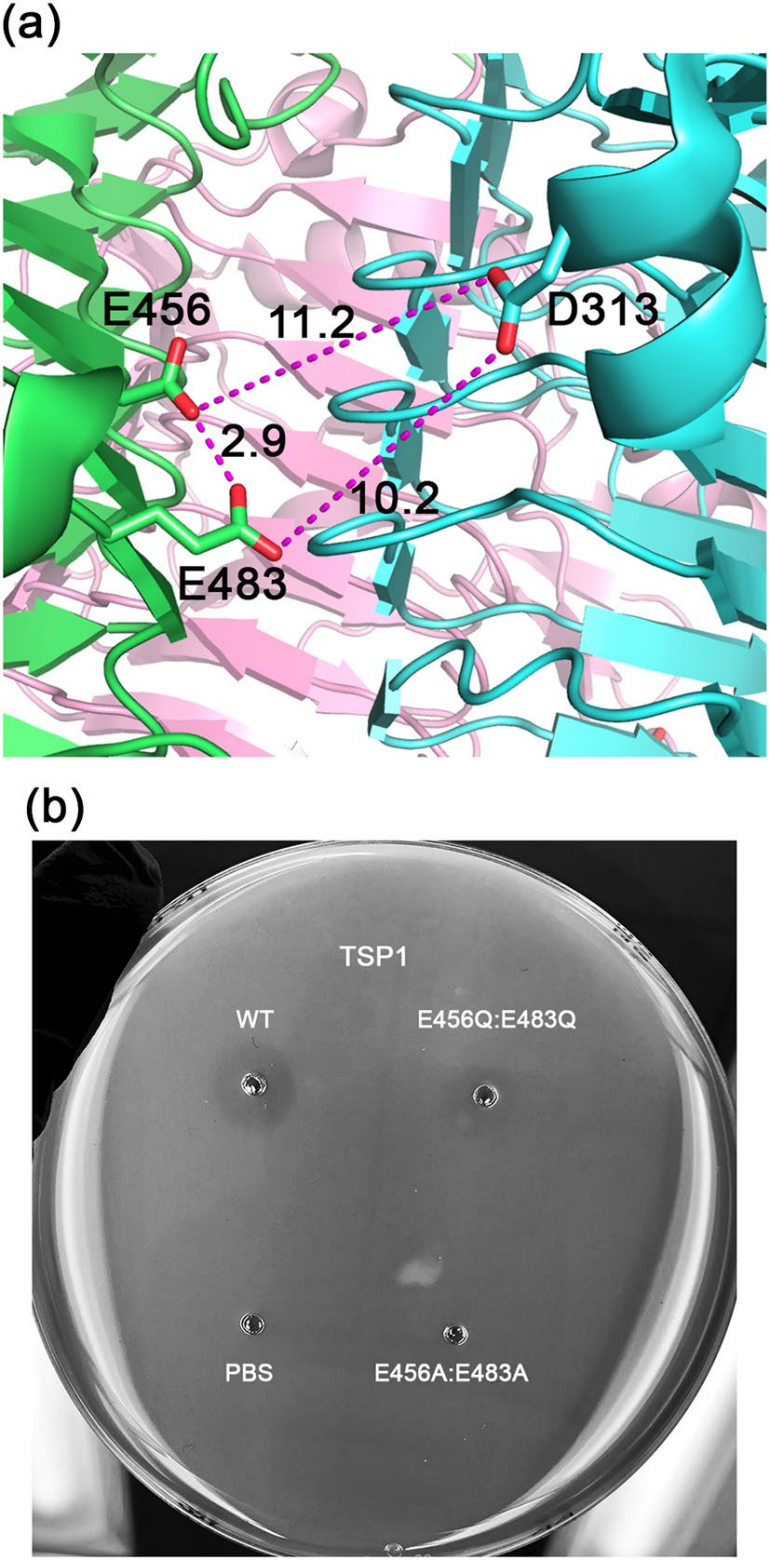
TSP1 active site structure and halo assay of active site residues. Structure and mechanism-based analyses were used to identify the active site residues. (**a**) Depiction of key carboxylic acid residues in the interface between two TSP1 subunits. The figure was generated using the computer program PyMol v1.8.0.2 (Schrödinger, LLC, https://www.pymol.org). (**b**) Halo assay of wild-type and mutant TSP1. *E. coli* strain ATCC 700728 was embedded in agarose. Wells (3 mm) were cut out of the agarose and loaded with 10 µL (6 mg/mL) of active site TSP1 mutants, and incubated overnight at 37 °C to visualize glycosidase activity. The absence of a halo for E456A:E483A TSP1 suggests an inhibition of enzymatic activity. Alternatively, the appearance of a halo for the E456Q:E483Q mutant indicates TSP1 retains the ability to display glycosidase activity. Wild-type TSP1 and PBS only served as positive and negative controls for glycosidase activity, respectively.

We previously proposed that the active site of TSP1 is located within the intermolecular cleft, and that Glu456 and Glu483 comprise the TSP1 catalytic machinery^9^. These are two adjacent intramolecular glutamic acids whose carboxylic groups are positioned 2.9 Å apart, therefore they are likely to share a proton (Fig. 9a). A similar arrangement is observed for glycosidases that act by the substrate-assisted mechanism proposed for glycosyl hydrolases belonging to the chitinolytic enzymes of families 18, and 20 [36, 37, 38], and to the hyaluronidases of family 56 [39, 40]. At the time, we could not confirm this proposal experimentally because of lack of an assay to detect glycosidase activity^9^. As can be seen in Fig. 2, TSP1 produces a halo, which enabled the probing of the proposed catalytic residues. All TSP1 mutants could be purified as soluble trimeric proteins. Reminiscent of TSP2, E456A:E483A TSP1 exhibited no halo whereas E456Q:E483Q TSP1 generated a halo (Fig. 9b). Yet, in contrast to TSP2, TSP1 does not contain obvious alternative glycosidase catalytic machinery. Asp313 on the adjacent TSP1 subunit is located 9.3–10.2 Å away from Glu483 and 10.8–11.3 Å away from Glu456 and could conceivably be part of an alternative acid/base pair (Fig. 9a). Unfortunately, the triple mutant D313N:E456Q:E483Q TSP1 produced heavy white aggregate, which impeded halo detection (data not shown). Two tyrosine residues flank the catalytic Glu456 and Glu483. However, both tyrosine side chains are located remotely from Asp313 and there is no positive charge sufficiently close to either of them that could modulate the hydroxyl pKa. Hence, the origin of the catalytic activity of the E456Q:E483Q TSP1 remains unclear.

## Conclusion

In conclusion, the crystal structure of full-length TSP2 reveals a head domain comprised only of the N-terminal fold module (D1) seen in three CBA120 TSPs but lacking the D2 fold module. The 168-residue long N-terminal region is disordered in the crystal and may require interactions with other TSP partners for adopting a unique three-dimensional structure. The crystal structures of TSP1, TSP2, and TSP3 suggest an active site location within the intermolecular crevice and catalytic machinery involving two carboxyl groups unique to each protein. Holo assays show that these TSPs can degrade polysaccharides on the surface of *E. coli* O157:H7 even in the absence of O-antigen, facilitating the identification of the catalytic residues using site directed mutant proteins. The identities of the catalytic residues of TSP3 were reported previously. Herewith, site-directed mutagenesis coupled with the halo assays confirm the structure-based hypotheses for TSP1 and TSP2. We show that elimination of activity requires replacement of both carboxylic acids by alanine residues whereas the enzyme remains active when only one carboxylic acid is eliminated. Moreover, replacement of the catalytic Asp/Glu by Asn/Gln does not eliminate catalytic activity. In TSP1, as proposed earlier^9^, two interacting glutamic acids residues of TSP1 facilitate a substrate-assisted glycosidase mechanism, whereas the catalytic mechanism of TSP2 relies on two protein carboxylic acids. The environment of the catalytic machinery of each TSP is different, and the mutant analysis underscores the variations in the acid/base mechanism. Strikingly, the mutagenesis studies highlight an interesting feature of the TSP2 active site architecture, whereby an alternative catalytic apparatus is unmasked when the two catalytic carboxyl groups are replaced by amide groups. This may be relevant to the evolution of glycosidase mechanisms in TSPs, and for the evolution and optimization of catalytic mechanisms in general.

## Materials and methods

### Cloning, expression and purification

The nucleic acid sequence of TSP2 was codon optimized for expression in *E. coli*, modified to add a C-terminal 6X-His tag, and synthesized by GeneArt (Invitrogen). The genes were sub-cloned into a pBAD24 expression vector, and the resulting clones were used to transform Rosetta-gami 2 Competent Cells^24^. The transformed cells were used to inoculate 2 × yeast extract tryptone (YT) medium supplemented with 200 μg/mL ampicillin at 37 °C for 4–8 h until the optical density at 600 nm (OD_600_) reached 0.8. The temperature was lowered to 18 °C for induction with 0.25% (w/v) l-arabinose and the biomass was collected after overnight growth by centrifugation. For each TSP, the pellet was resuspended in phosphate buffered saline (PBS), pH 7.4, lysed by sonication, and centrifuged at 14,000 rpm for 45 min. The resulting supernatant was incubated with Ni–NTA agarose for 1 h at 4 °C. The TSPs were purified by Ni affinity gravity-flow chromatography, and dialyzed in Tris–HCl buffer (pH 7.5). The cloning, expression, and purification of TSP1 and TSP3 were reported previously^9,10^. The mass, purity, and solubility of all wild type TSPs and mutant TSPs were assessed by SDS-PAGE.

### Site-directed mutagenesis

Plasmids harboring the TSP1 and TSP2 mutants were constructed using the Q5 Site-Directed Mutagenesis Kit (New England Biolabs) as described previously^10^. Primers were designed to include mutations in the middle of 30 nucleotide forward primers, with a universal *Amp* reverse primer. The resulting PCR products were digested by *DpnI* to remove the methylated templates, ligated and transformed into *E. coli* DH5ɑ. The mutations were confirmed by nucleotide sequencing (Macrogen, USA) before being transformed into *E. coli* BL21(DE3) for protein expression. Mutants were purified similar to wild type TSPs and all recombinant proteins were soluble and stable.

### Analytical size-exclusion chromatography

The multimeric state of recombinant TSP2 was determined by analytical size-exclusion chromatography as reported previously^9,10^. The protein was applied to a pre-equilibrated Superose 6 column (GE Healthcare) and run under isocratic conditions in PBS for 1.5 column volumes on an AKTA FPLC system (GE Healthcare). The molecular mass of TSP2 was estimated from a standard curve generated by linear regression of log (molecular mass) vs. retention volume using gel filtration standards (Bio-Rad).

### Thermal stability measurements

The thermal stability of TSP2 was investigated using a Chirascan CD Spectrometer (Applied Photophysics) following the same protocol as reported previously^9,10^. TSP2 (0.1 mg/mL) in 20 mM sodium phosphate buffer (pH 7.0) was gradually heated at a rate of 1 °C/min from 20 °C to 95 °C. The mean residue ellipticity of the sample contained in a quartz cuvette of 1 mm path length was monitored every 0.5 °C at 218 nm with 5 s signal averaging per data point. The T_m_ was calculated using the Pro-Data software (Applied Photophysics) based on data that was fitted to a Boltzmann sigmoidal curve.

### Crystallization and structure determination

Crystals of wild-type TSP2 were obtained by the vapor diffusion method in sitting drops at room temperature. The reservoir solutions contained 0.8 M ammonium sulfate and 0.1 M HEPES (pH 7.0). A couple of crystals useful for diffraction data collection appeared after five weeks and could not be reproduced despite much subsequent effort. The crystal was cryoprotected by adding to the drop equal volume of reservoir solution supplemented with 30% (v/v) glycerol, transferring the crystal to a mounting pin and flash cooling in liquid nitrogen. X-ray diffraction data were collected at beamline 23-ID_B managed by the General Medical Sciences and National Cancer Institute collaborative access team (GM/CA-CAT) at Argonne National Laboratory. The beamline was equipped with a MARmosaic 300 CCD detector (Marresearch GmbH). Diffraction data was acquired at 1.0332 Å. The diffraction data extended to a resolution of 1.9 Å. The data was processed using XDS^25^ and Aimless^26^. The TSP2 catalytic domain crystal structure^6^ provided the search model for structure determination by Molecular Replacement using Phaser^27^ as implemented in Phenix^28^ and refined with Refmac^29^ and Phenix Refine^30^. Structure modification was carried out using the interactive graphics computer program COOT^31^. Structure figures were prepared using the program PyMol (Schrödinger, LLC).

### Halo assay

Halo assays were performed following the same protocol described previously^10^. For routine assays, a non-toxigenic strain of *E. coli* O157:H7 (ATCC 700728) was used. Bacterial strains were grown overnight at 37 °C with aeration to an OD_600_ = 1.6. After overnight growth, the bacterial cells were harvested via centrifugation at 4,150 rpm for 10 min at 4 °C. The cell pellets were washed twice using sterile PBS buffer and resuspended in buffer at 1/50 of the original volume. Next, 500 µL of concentrated bacterial cells were mixed with 10 mL of sterile 0.7% (w/v) agarose solution and plated in a disposable Petri dish (Fisher Scientific). Holes (wells) with diameters of ~ 3 mm were generated on the solidified agarose using sterile plastic dropper tips (Fisher Scientific). 10 µL of either wild-type TSP or its active site mutants were added to each hole at a concentration of 6 mg/mL. Petri dishes were incubated for 24 h at 37 °C. Halos were visualized by holding the Petri dish to a light box and photographing with a 12-megapixel camera (iPhone 6S Plus). Contrast and brightness adjustments were applied to the entire image using Photoshop CC (Adobe, Inc.) Clearing zones (darker areas compared with intact agar-embedded bacteria) correspond to glycosidase activity.

### Turbidity assay

*Escherichia coli* O157:H7 (ATCC 700728) culture was grown at 37 °C to OD_600_ of 0.2. 300 µL bacterial culture was mixed with 100 µg/mL, 50 µg/mL, 25 µg/mL, 10 µg/mL or 5 µg/mL (final concentration) of TSPs in SM buffer. The mixture was incubated for 15 min at 37 °C. 150 µL phage CBA120 at concentration of 6 × 10^9^ PFU/mL in SM buffer was added to the *E. coli* and TSP mixture. Control solutions included *E. coli* alone (uninterrupted bacterial growth), *E. coli* and phage without TSP (maximum phage activity), and *E. coli* with TSP in the absence of phage (confirmation that the TSP does not interfere with bacterial growth). 180 µL of each mixture was transferred into the 96 well plates in technical triplicate. The plates were incubated at 37 °C, and the OD_600_ was measured every 20 min for 15 h.

## Data Availability

The coordinates and structure factors were deposited in the Protein Databank, entry code **6W4Q.**
